# Laparoscopic resection of a giant retroperitoneal melanotic schwannoma

**DOI:** 10.1093/jscr/rjy040

**Published:** 2018-03-26

**Authors:** Florian Chatelet, Armelle Bardier-Dupas, Fabrice Menegaux, Nathalie Chereau

**Affiliations:** 1 Department of General and Endocrine Surgery, Hospital Pitié Salpêtrière, APHP Pierre et Marie Curie University, Sorbonne Universities, Paris, France; 2 Department of Pathology, Hospital Pitié Salpêtrière, APHP Pierre et Marie Curie University, Sorbonne Universities, Paris, France

## Abstract

**Background:**

Retroperitoneal schwannomas are extremely rare, as they account for only 3% of retroperitoneal tumors. Clinical symptoms are nonspecific and of late onset, meaning that these tumors are diagnosed at an advanced stage. Surgical resection is required for histological diagnosis and to prevent possible malignant transformation. Celioscopy offers numerous benefits, reducing postoperative pain and speeding up the patient’s return to autonomy, but it can pose a real challenge due to the size of these lesions.

**Case presentation:**

We report a case of laparoscopic resection of a very large right-sided retroperitoneal schwannoma, with a particular histological form.

**Conclusion:**

Surgical resection in a single unit remains the golden rule, and a laparoscopy can be proposed when the diagnosis is beyond doubt. The large size of the retroperitoneal melanotic schwannomas, a common feature, increases surgical difficulties but is not a contraindication to this approach.

## INTRODUCTION

Retroperitoneal schwannomas are extremely rare, as they account for only 3% of retroperitoneal tumors [1–4]. Clinical symptoms are nonspecific and of late onset, meaning that these tumors are diagnosed at an advanced stage. Surgical resection is required for histological diagnosis and to prevent possible malignant transformation. Celioscopy offers numerous benefits, reducing postoperative pain and speeding up the patient’s return to autonomy, but it can pose a real challenge due to the size of these lesions.

## CASE REPORT

**Figure 1:**
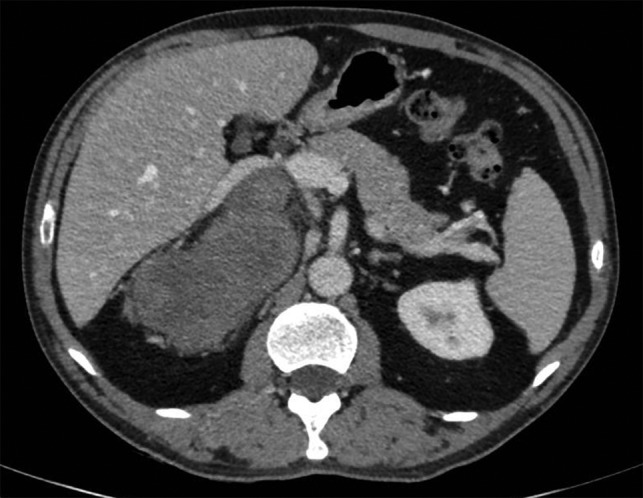
CT imaging of the lesion.

A 57-year-old man was admitted following the accidental discovery of a large mass in the right hypochondrium, detected during an ultrasound examination performed in the context of an assessment of urethrorrhagia. When questioned, the patient reported, with hindsight, upper body cyanosis in anteflexion. A CT scan showed a right-sided retroperitoneal mass measuring 100 × 165 × 164 mm, pushing the liver and right adrenal gland forward, with the inferior vena cava narrowed and pushed forward in the same way (Fig. 1). A cleavage plane was described with all the adjacent structures (liver, kidney, inferior vena cava). The mass was encapsulated, well circumscribed, trilobed, of tissular appearance, with spontaneous density of 35 UH, heterogeneous, containing liquid portions of necrotic appearance and two punctiform calcifications. After injection of the contrast product, there was moderate and heterogeneous uptake of the mass in late portal time (+10 min).

**Figure 2:**
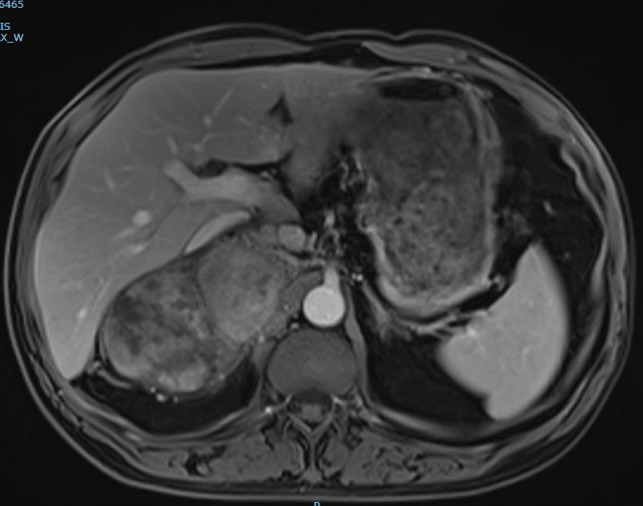
T1-weighted MRI image with gadolinium injection.

**Figure 3:**
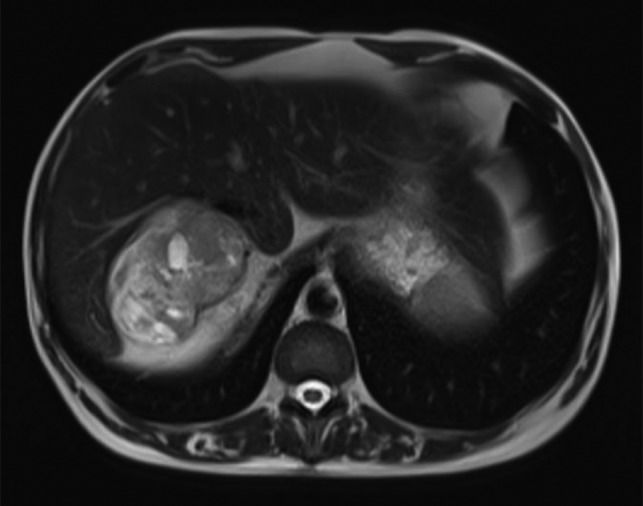
T2-weighted MRI image.

**Figure 4:**
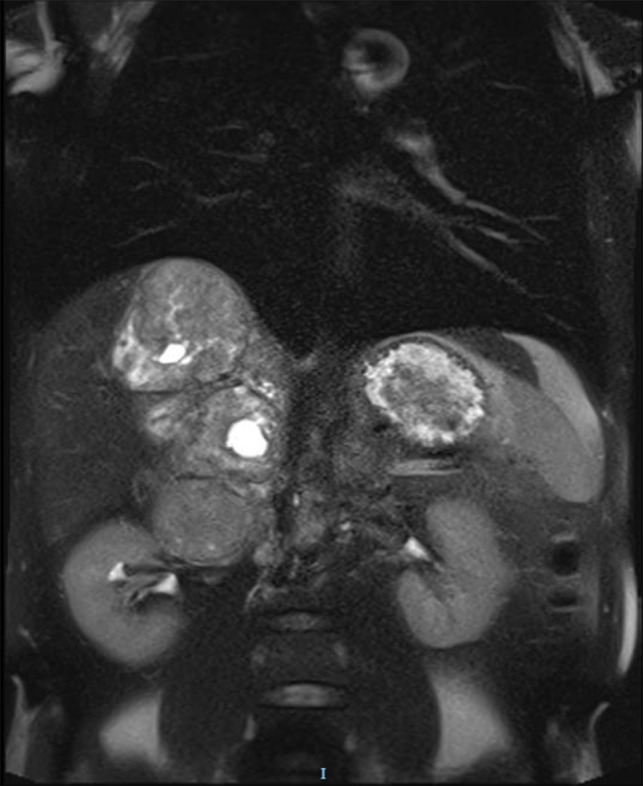
T2-weighted MRI coronal plane.

The magnetic resonance imaging (MRI) found the mass, which was measured at 85 × 60 × 160 mm, heterogeneous, with isointense and T1-weighted hyposignal (Fig. 2), isointense and T2-weighted hypersignal (Figs 3 and 4), and diffusion-weighted heterogeneous hypersignal. After injection of the contrast product, the mass did not appear to be hypervascular, and uptake was more pronounced in the late phase, indicative of a fibrous nature. The FDG-PET scan showed a hypermetabolic mass with SUVmax of 6.3. Finally, after excess secretion of catecholamines had been ruled out, a CT-guided biopsy was performed, with the histological analysis concluding that a schwannoma was highly probable.

The patient had celioscopic surgery in the left lateral decubitus position under general anesthesia, with a 30° camera inserted into a right pararectal trocar (open celioscopy), then three other trocars (5 mm) under scope control, two in the right side and one in the right sub-costal region on the pararectal line.

The latter was completely mobilized as far as the subhepatic veins by sectioning the triangular ligament, and the inferior vena cava and right renal vein were then identified and separated from the mass. The adrenal gland was resected as a single unit in order to avoid the risk of tumor rupture. A short sub-costal laparotomy, necessary to extract the tumor, was performed to make it possible to lift the mass up by placing one hand in the wound, with the wall being protected by a plastic retractor ring. The postoperative period was straightforward, with the patient taking food the next day, while the drain was removed on Day 2 and the patient returned home on Day 4.

**Figure 5:**
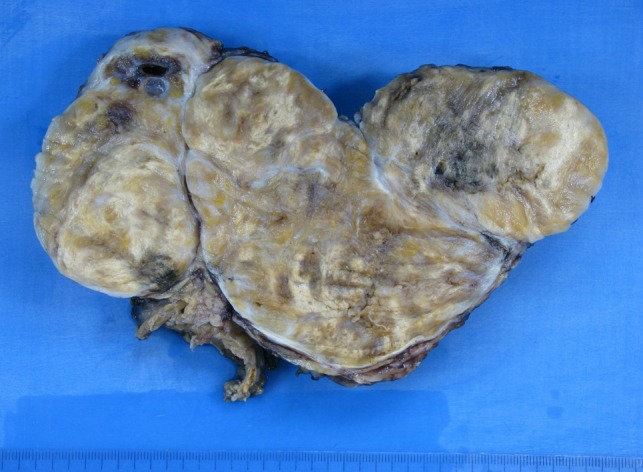
Histopathology.

The histological examination showed that the mass weighed 618 g and measured 17 × 8 × 6 cm. It had a lobular, nodular, well circumscribed, encapsulated, heterogeneous appearance, yellow or beige with necrotic black areas (Fig. 5). Histologically, this was a melanotic schwannoma, with immunohistochemical tests showing diffuse positivity for SOX10 and PS100, CD34 and CD117 markers were negative, and MelanA marked cytoplasmic pigment in some tumor cells. There was one mitosis for 10 fields with G40, the resection margins were in healthy tissue and the adrenal gland was normal.

## DISCUSSION

Retroperitoneal melanotic schwannomas are extremely rare: to the best of our knowledge, only four cases had been reported until our case study [5–8].

They may be seen in the context of Carney complex, a hereditary syndrome comprising myxomas (cardiac, skin or breast), skin pigmentation and endocrine hyperactivity (dysthyroidism, pituitary adenomas, Cushing’s syndrome) [9]. There was nothing to suggest Carney complex in the case reported here. Melanotic schwannomas are traditionally considered to be benign, slow-growing tumors, but 15–42% of patients will suffer a recurrence [5, 9, 10].

Treatment consists of total excision of the lesion, taking care to avoid tumor effraction [10]. If a case of retroperitoneal melanotic schwannoma is diagnosed preoperatively, as in our case study where imaging tests raised the possibility and the diagnosis was confirmed by biopsy, a laparoscopic approach can be suggested first for excision [1] despite the fact that the tumor is often very large. The main advantages of laparoscopy are a reduction in perioperative bleeding, postoperative pain and time in hospital, and the esthetic benefit. The intervention can be started in celioscopic mode as the dissection planes are generally very well delineated, but we must accept the risk of being forced to change to laparotomy in the event of technical difficulties, hemorrhage, or to avoid any risk of tumor rupture.

## CONCLUSION

Retroperitoneal melanotic schwannomas are extremely rare. Surgical resection in a single unit remains the golden rule, and a laparoscopy can be proposed when the diagnosis is beyond doubt. The large size of the tumor, a common feature, increases surgical difficulties but is not a contraindication to this approach.
